# To use or not to use a condom: A prospective cohort study comparing contraceptive practices among HIV-infected and HIV-negative youth in Uganda

**DOI:** 10.1186/1471-2334-11-144

**Published:** 2011-05-23

**Authors:** Jolly Beyeza-Kashesya, Frank Kaharuza, Anna Mia Ekström, Stella Neema, Asli Kulane, Florence Mirembe

**Affiliations:** 1 Department of Obstetrics/Gynaecology, Makerere University College of Health Sciences, Kampala Uganda; 2 Division of Global Health, Department of Public Health Sciences, Karolinska Institutet, Stockholm, Sweden; 3 Makerere University Institute of Social Research, Kampala Uganda

## Abstract

**Background:**

Unwanted pregnancy and HIV infection are issues of significant concern to young people. Limited data exists on contraceptive decision-making and practices among HIV-infected and HIV-negative young people in low resource settings with generalized HIV epidemics.

**Methods:**

From July 2007 until April 2009, we recruited, and followed up over a one year period, a cohort of 501 HIV-negative and 276 HIV-infected young women and men aged 15-24 years residing in Kampala and Wakiso districts. We compared contraceptive use among HIV-infected and HIV-negative young people and assessed factors associated with contraceptive decision-making and use, using multivariate logistic regression modelling to estimate odds ratios (OR) and 95% confidence intervals (CI).

**Results:**

Contraceptive use among sexually active HIV-infected young people was 34% while it was 59% among the HIV-negative group. The condom was the most frequently used method of contraception. Only 24% of the HIV-infected used condoms consistently compared to 38% among the negative group OR 0.56 (95% CI 0.38, 0.82). HIV-infected young people were more likely to discuss safe sex behaviour with health workers OR 1.70 (95% CI 1.13, 2.57), though its effect on fertility decision-making was not significant. Throughout the year's follow-up, only 24% among the HIV-negative and 18% among the HIV-infected continued to use contraception while 12% and 28% among the HIV-negative and infected respectively did not use contraception at all. At multivariate analysis, the HIV-infected young people were less likely to maintain contraceptive use. Other factors independently associated with sustained contraceptive use were age of the respondent, marital status and being a male. Conversely, HIV-infected young people were less likely to initiate use of contraception. Being married or in a relationship was associated with higher odds of initiating contraceptive use.

**Conclusion:**

Compared to the HIV-negative group, sexually active HIV-infected young people are less likely to use contraception and condoms. Initiating or sustaining contraceptive use was also significantly less among the HIV-infected group. Strengthening family planning services and developing new innovative ideas to re-market condom use are needed. Policy and guidelines that empower health workers to help young people (especially the HIV infected) express their sexuality and reproduction should urgently be developed.

## Background

Unwanted pregnancies and HIV infection continue to be daunting problems for young people, and studies indicate that HIV-infected youth face the greatest dilemmas [1]. Globally, young people aged between 15 and 24 years make up 1.2 billion of the world's population. The majority live in Sub-Saharan Africa and are vulnerable to unwanted pregnancies and HIV infection [2,3].

### Monitoring HIV prevalence among young people is a good proxy indicator for HIV incidence in a country

In Uganda, the HIV prevalence among men and women aged between 15 to 17 years is 0.3% and 1.9%, and higher at 2.3% and 5.5% among 20-24 year old men and women indicating a gender vulnerability and disparity in sexual practices especially when girls engage in cross-generational sex with older men often with compromised negotiation for safe sex [4]. Early sexual activity and lack of access to contraception leads to a high unmet need for contraception and poor reproductive health indices among Ugandan youth. Only 5% of sexually active young people aged 15 to 19 years and 18% aged 20 to 24 year old used contraception, leading to very high rates (40%) of unintended and often unwanted pregnancies among the adolescents [3].

Abortion is illegal in Uganda but the abortion rate is about 54 per 1000 women aged between 15-49 years, and possibly there are similar rates among adolescents [3,5,6]. Abortion is estimated to contribute to 15-30% of maternal deaths in developing countries most of which are adolescents [7-9]. Furthermore, young people face enormous sexual and reproductive health challenges when seeking reproductive health information and services [10,11]. A lack of youth-friendly services, stigma associated with premarital sex, STI/HIV/AIDS, adolescent pregnancy, non-disclosure of HIV status, and the health workers' attitude all play a big role in determining access to reproductive health care by young people [10,12-15].

Management of sexual and reproductive health of HIV-infected youth is critical to reducing HIV transmission and maternal mortality. While unwanted pregnancies among adolescents contribute to a high maternal mortality in Uganda, overwhelming evidence shows that HIV infection is also associated with increased maternal mortality and morbidity [16-19]. Moreover, prevention of mother to child transmission of HIV is still a major challenge because effective combination antiretroviral therapy (ART) only reaches a minority of HIV-infected women [4], and the access to skilled attendance at birth is still sub-optimal [3]. Little information is available on reproductive practices among HIV-infected young people that could inform the design of new and culturally appropriate interventions. We explored contraceptive decision-making and practices among HIV-infected and negative youth over a period of one year.

## Methods

### Study design and setting

From July 2007 to April 2008, we recruited a cohort of 15-24 year old HIV-infected and negative young people. Each participant was followed up for one year. The HIV-infected group were identified from TASO Mulago; a unit that provides care for HIV/AIDS clients [20]. The HIV-negative group were recruited from Naguru Teenage and Information Centre (NTIC); a unit that offers youth-friendly services to young people aged 10 to 24 years. Both clinics are located in Kampala, Uganda's capital city, and serve both urban and rural young people in Kampala and its surrounding communities.

### Participant recruitment

All study procedures were conducted at the end of a clinic visit. At TASO, the registered clients go through counselling, medical consultations, laboratory diagnostics if required, and get antiretroviral treatment if indicated before going home. A specific counsellor was assigned to work with the study team to identify the young people and referred them to the study team to screen for study eligibility. Young people, who knew their HIV status (negative or positive) for at least six months before the recruitment date, were resident within a radius of 30 km of the clinic, and were not intending to relocate within two years were eligible for the study. The study protocol was explained to eligible respondents and informed consent for study participation obtained. All eligible HIV-infected young people were then consecutively recruited until the required sample size was reached.

Of 60-80 clients attending NTIC every day 50% are new clients and 25% have never tested for HIV infection. HIV-negative young people who were resident within 30 Km of NTIC, and had been tested for HIV at least six months before study date were eligible for study. The HIV-infected young people attending NTIC were excluded from the study to avoid double recruitment since NTIC does not offer specified HIV care and refers those needing antiretroviral treatment to other units like TASO or IDC, but nevertheless they keep coming to NTIC for other services". A systematic random sampling of eligible HIV-negative young people was used. We used the clinic register to identify the eligible clients. Every second client was approached for possible recruitment. If they declined participation, the next person on the register would then qualify for recruitment.

### Sampling and data collection

The sample size was calculated using the standard formula for cohort studies [21]. To detect a 10% contraceptive prevalence difference between the HIV-infected and HIV-negative groups, contraception prevalence among sexually active women (15-49 years) of 18% was used [3]. At an 80% power and 95% confidence level, allowing for 15% loss to follow up, and ratio of 1:2, we needed to recruit 250 HIV-infected and 500 HIV-negative young people.

Data were collected using a structured survey questionnaire with closed and open-ended questions. Socio-demographic characteristics, symptoms of HIV (infected youth), fertility decisions (desire for pregnancy, time of when to get pregnant and contraceptive use), and discussion with partner (for those who had partners) and extended family and health care workers were asked. The main outcomes of the study were desire for children, condom use and contraceptive use (both uptake and discontinuation of contraception, sustained contraceptive use and sustained non-use). Study participants were interviewed at baseline, six months, and twelve months after recruitment.

### Identification of respondents for follow up

A contact locator form with address and telephone numbers of respondent and/or their head of household or guardian, a relative, spouse or friend were recorded. A small card with their particulars and unique identification number was given to the respondent for presentation at the follow-up visit. This information helped to locate respondents who did not return for their scheduled appointment. A small transport subsidy of 1.8$ to 2.5$, depending on journey distance from home, was given to each respondent on each visit. Study staff contacted the participants by phone or home visited within 4 weeks of a missed appointment. Clients were considered lost to follow up if they did not participate in an interview within three months of a scheduled visit and after four telephone calls and a home visit.

### Data Management and Analysis

Data were double-entered using Epi Info cleaned, coded and analysed using STATA 10. All the data analysed compared the HIV-negative and HIV-infected population. The outcomes of interest were desire for children, condom use and contraceptive use (both uptake and discontinuation). Explanatory factors studied were the socio-demographic characteristics, discussion with partner about fertility, and having discussed with health workers about pregnancy, contraception and HIV. Descriptive statistics to compare the baseline characteristics of HIV-infected and HIV-negative young people were done using chi-square test for categorical data and Student t-test for continuous data.

Condom use responses ("always", "sometimes" and "never") were dichotomised into a new variable with always as consistent use and "sometimes and never" merged to inconsistent use. Responses to the question "If 100 HIV-infected people used the condom, how many would not get HIV transmitted to the partners?" were used to assess knowledge of condom effectiveness. Furthermore, a composite variable of "discussion with health workers" was generated from three variables of discussion about pregnancy, contraception and birth spacing. Similarly, "discussion with partner about childbearing" was generated from discussion about the number of children to have and when to have them.

To assess the dynamics of contraceptive use during the one year of follow-up, four sub-analyses were done for participants who: initiated contraceptive use, discontinued contraceptive use, continued to use contraception and those who did not use contraception at all. We considered changes in the four outcomes at six months interval. This aimed to assess factors that influence either continued contraceptive use or non-contraceptive use. For this study, contraceptive uptake was defined as initiation of contraceptive use at 6 and 12 months after baseline. We considered that clients who were sexually active were eligible to use contraception and made the denominator. At the six-month follow-up, all sexually active clients who were not using contraception at baseline plus those who had been sexually abstinent at baseline but became sexually active in that period were recorded as having initiated contraceptive use (Contraceptive uptake) if they reported they were using contraception. At the twelve-month follow-up, a similar consideration was done. All sexually active clients who were not using contraception at the six-month follow-up were recorded as up-takers of contraception if they answered that they were currently using contraception. Therefore, a person could contribute both to uptake in the first six months if he/she started using contraception, and to discontinuation later in the year is he/she stopped the contraception.

Contraceptive discontinuation was defined as stopping the use of contraception during the 12 months after baseline or after initiation of use at any time during the follow-up. At the six-month follow-up, all respondents who were using contraception at baseline but had stopped contraceptive use we considered "discontinued contraception". At the twelve-month follow-up, all respondents who were using contraception at six months but had stopped contraceptive use were considered as discontinued contraception. Consistent contraceptive use was defined as continued use at baseline, six months and twelve months. Similarly, consistent non-use was defined as non-use at baseline, six and twelve months.

The study protocol was approved by the Makerere University, College of Health Sciences Higher Degrees Research and Ethics Committee, the Uganda National Council of Science and Technology, and the institutional review board of TASO. All participants provided informed consent.

## Results

**Figure 1:**
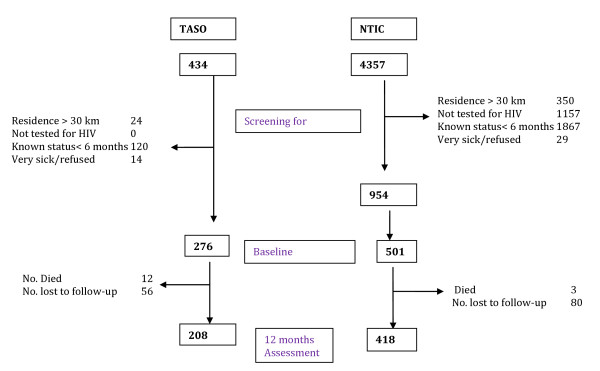
Flowchart for patient recruitment and follow-up

Of the 4,357 young people screened for eligibility at NTIC, 954 were found eligible, and 501 were recruited. At TASO, 434 were screened for eligibility and 276 were recruited (Figure 1). Social demographic characteristics of the HIV-infected and HIV-negative groups were similar except for education and marital status (Table 1). HIV-infected young people were less likely than HIV-negative youth to be in school, had no or only primary education (43%), had had more life time sexual partners, and had older partners. Furthermore, more HIV-infected young people were likely to be in polygamous relationships. Slightly more than half (52%) of the HIV-infected group and 18% among HIV-negative group had had children. The mean number of living children was 1.43 children (SD, 0.79) among the HIV-infected youth and 0.78 children (SD, 0.64) among the HIV-negative youth. Eleven percent of the HIV-negative group and five percent of the HIV-infected group were pregnant at the time of recruitment into the study. About two percent in both groups were not sure if they were pregnant.

**Table 1:** Socio-demographic characteristics of HIV-infected and negative young people

Characteristics	Negative N = 501	Infected N = 277	P value
Age of respondent	N (%)	N (%)	
Age 15 - 19 years	203 (40.1)	116 (41.9)	
Age 20 - 24 years	298 (59.5)	161 (58.1)	0.712
Median age (IQR)	20 (18-21)	21 (17-23)	
Gender of respondent			
Male	139 (27.7)	61 (22.0)	0.080
Female	362(72.3)	216 (78.0)	
Religion			
Catholic	174 (34.9)	90 (32.5)	
Protestant	165 (33.1)	77 (27.8)	
Muslim	60 (12.1)	49 (17.7)	
Born again Christians/others	99 (19.9)	61 (22.0)	0.095
Are you in school?			
Yes	280 (56.1)	102 (37.0)	0.000
Education level			
0-7 years	67 (13.4)	120 (43.3)	
8 -11 years	181 (36.3)	126 (45.5)	
12 + years	251 (50.3)	31 (11.2)	0.000
Marital status			
Single	142 (28.3)	110 (39.9)	
Married/in a relationship	348 (69.5)	131 (47.5)	0.000
Separated or widowed	11 (2.2)	35 (12.7)	
Duration of relationship			
Less than three years	145 (40.5)	72 (53.30	
Three years and more	213 (59.5)	63 (46.7)	0.010
Type of relationship			
Monogamous	328 (91.9)	114 (82.0)	0.002
Polygamous	29 (8.1)	25 (18.0)	
Median duration of relationship (IQR)*	2(1- 3)	3 (1 - 4)	0.033
Partner's age difference (IQR)*	4 (2-6)	5 (3-9)	0.000
Number of life time sexual partners (IQR)*	2 (1- 3)	3 (1 - 4)	0.009
Number of current sexual partners*	1(1-1)	1 (1-1)	0.155
Have Children?			
Yes	89 (18.0)	131 (52.2)	0.000
Number of living children mean (SD)	0.78 (±0.64)	1.43 (±0.79)	0.000

*P values -used non-parametric methods

### Contraceptive use

**Figure 2:**
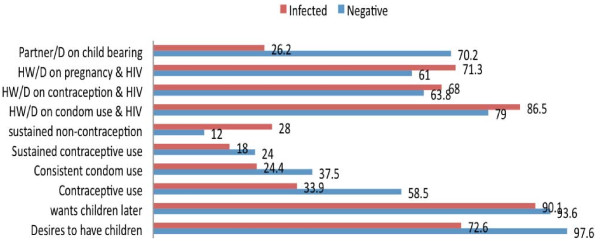
**Contraceptive behaviour among HIV-infected and negative young people at baseline and 12 months follow-up**. Note: Partner/D on child bearing - Discussed with partner on child bearing. HW/D on pregnancy & HIV - Discussed with H/workers about pregnancy & HIV. HW/D on contraception & HIV - Discussed with H/Workers about contraception & HIV. HW/D on condom use & HIV - Discussed with H/workers about condom use & HIV

Figure 2 describes the contraceptive behaviour of a sub-sample of the sexually active young people in the cohort. Desire for children was 72% among the HIV infected and 98% among the HIV-negative group. Of those who desired children, more than 90% in both groups wanted to have them within four years of the time of interview. Sexually active HIV-infected young people were less likely to use contraception compared to the HIV-negative group. Pvalue = 0.000. The most commonly used method of contraception in both groups was the condom i.e. 77% (72% among the infected youth and 79% among the negative youth). Other methods included using pills or injections: 19% among the HIV-negative and 21% among the HIV-infected youth. Only 2% of HIV-negative and 6% of the HIV-infected young people used the recommended dual methods. Consistent condom use was very low among this group with the HIV-infected group (24%) less likely to consistently used condoms compared to the HIV-negative group (38%), p value = 0.002. A significant minority (39%) among the HIV-infected had never used condoms compared to 17% among the HIV-negative. HIV-infected young people were less likely to use condoms with all sexual partners p value = 0.000; and at the last sexual act, p value = 0.019.

Knowledge of condom effectiveness was lower among the HIV-infected compared to the HIV-negative. The HIV-infected young people thought using a condom was only 60% effective in preventing HIV transmission (IQR; 40-95), while the HIV-negative group considered the condom was 80% effective (IQR; 50-99) (t-test p = 0.007). However, discussion with health workers about contraception was similar in both groups. (Figure 2)

### Trends in contraceptive use among young people

During the one-year follow-up period, the HIV-infected group were less likely to consistently use contraception compared to HIV-negative group (OR 0.63 (0.41, 0.98), p-value = 0.038); were less likely to start using contraception (contraceptive uptake) than the HIV-negative young people (OR 0.63, (0.44, 0.91), p-value = 0.012). In addition, contraceptive uptake was significantly greater among the young people who were married or were in a relationship compared to sexually active singles, (OR 2.46 (1.68, 3.62), p-value = 0.000), among the 20 to 24 year olds (OR 1.73 (1.22, 2.46), p-value = 0.002), and among those who discussed with their partner (OR 1.81 (1.28, 2.57), p-value = 0.001). However, there was no difference in contraceptive uptake between those who discussed with health workers about contraception, pregnancy or birth spacing and those who did not. Nonetheless, there was no difference in discontinuing contraceptive use among the two groups (OR 0.73 (0.44, 1.22), p-value = 0.223). (Table 2)

**Table 2:** Trends in contraceptive use (uptake and discontinuation) among young people

	Contraceptive use at Baseline			Discontinued use during 12 months follow-up			Uptake during 12 months follow-up			
**Factors**	**Total 600**	**N (%)**	**P value**	**Total 392**	**N (%)**	**P Value**	**Total 587**	**N (%)**	**P value**	**OR (95%CI)**

Age of respondent										
15- 19 years	214	79 (36.9)		117	73 (26.6)		266	75 (28.2)		1.0
20 -24 years	386	211 (54.7)	0.000	275	37 (31.6)	0.306	319	129 (40.4)	0.002	1.73 (1.22, 2.46)
HIV status of respondent										
Infected	248	84 (33.9)		110	26 (23.6)		215	61 (28.4)	0.012	0.63 (0.44, 0.91)
Negative	352	206 (58.5)	0.000	282	84 (29.8)	0.223	370	143 (38.7)		1.0
Sex of respondent										
Female	451	222 (49.2)		291	87 (29.9)		435	150 (34.5)		1.0
Male	149	68 (45.6)	0.448	101	23 (22.8)	0.170	150	54 (36.0)	0.737	1.07 (0.73, 1.58)
Marital status										
Single	156	24 (15.4)		55	19 (34.6)		235	55 (23.4)		1.0
Married/in a relationship	399	254 (63.7)	0.000	317	85 (26.8)	0.490	314	135 (43.0)	0.000	2.46 (1.68, 3.62)
Separated or widowed	45	12 (26.7)		20	6 (30.0)		36	14 (38.9)		2.08 (0.99, 4.37)
Education level										
Primary	162	75 (46.3)		88	22 (25.0)		136	38 (27.9)		1.0
Secondary	222	93 (41.9)		130	38 (29.2)		245	76 (31.0)	0.530	1.16 (0.73, 1.84)
Higher and above	214	121 (56.5)	0.008	173	49 (28.3)	0.780	202	89 (44.1)	0.003	2.03 (1.27, 3.26)
Age difference with partner										
5 years or less	239	159 (66.5)		202	57 (28.2)		193	84 (43.5)		1.0
6 years or more	128	78 (60.9)		93	26 (28.0)		98	43 (43.9)	0.954	1.01 (0.62, 1,66)
No partner	233	53 (22.8)	0.000	97	27 (27.8)	0.997	294	77 (26.2)	0.000	0.46 (0.31, 0.68)
Religion of respondent										
Born again (Pentecostal)	122	46 (37.7)		70	26 (37.1)		129	39 (30.2)		1.0
Catholic	211	107 (50.7)		131	34 (26.0)		193	60 (31.1)	0.871	1.04 (0.64, 1.69)
Protestant	180	91 (50.6)		130	35 (26.9)		184	73 (39.7)	0.087	1.52 (0.94, 2.46)
Muslim	84	43 (51.2)	0.085	58	15 (25.9)	0.341	77	30 (39.0)	0.200	1.47 (0.81, 2.67)
Discussed with health workers about childbearing										
No	68	33 (48.5)		48	10 (20.8)		87	30 (34.5)		
Yes	532	257 (48.3)	0.973	344	100(29.1)	0.234	498	174 (34.9)	0.937	1.02 (0.63, 1.65)
Discussed with partner about childbearing										
No	273	74 (27.1)		132	35 (26.5)		342	100 (29.2)		
Yes	327	216 (66.1)	0.000	266	75 (28.9)	0.627	243	104 (42.8)	0.001	1.81 (1.28, 2.57)

Note:

• Column labeled **contraceptive use at baseline **is the percentage of sexually active young people reporting using contraception at baseline stratified by various co-variates (the rows).

• Column heading "**discontinued use during the 12 months' follow-up**". This is includes those who were using contraception at baseline plus those who became sexually active, started use and discontinued by end of follow-up.

• **Uptake during the 12 months' follow-up**, the denominator comprised of those not using contraception at baseline including those who were abstaining but later started having sex.

In all, 107 (24%) HIV-negative and 37 (18%) HIV-infected continued to use contraception throughout the year, OR 0.63 (0.41, 0.98), while 45 (12%) and 67 (28%) among the HIV-negative and infected respectively did not use contraception at all, OR 2.80 (1.80, 4.36). At multivariate analysis, the HIV-infected were less likely to sustain contraceptive use. Other factors independently associated with sustained contraceptive use included: age of the respondent, marital status and being a male. Conversely, HIV-infected young people were more likely to not initiate contraceptive use (Table 3).

**Table 3:** Factors associated with sustaining contraceptive decision among young people

Factors	Sustained contraceptive use (N = 652)			Continued non-contraceptive use (N = 620)		
	**(Row %)**	**Crude OR**	**Adjusted OR**	**(Row %)**	**Crude OR**	**Adjusted OR**

Age of respondent	Yes			Yes		
15- 19 years	37 (15.2)	1.0	1.0	68 (24.7)	1.0	1.0
20 -24 years *	107 (26.2)	1.99 (1.31, 3.02)	1.78 (1.16, 2.74)	44 (12.8)	0.44 (0.29, 0.68)	0.85 (0.52, 1.37)
HIV status of respondent						
Negative	107 (24.1)	1.0	1.0	45 (11.8)	1.0	1.0
Infected	37 (17.8)	0.68 (0.45, 1.04)	0.63 (0.41, 0.98)	67 (28.2)	2.93 (1.91, 4.50)	2.80 (1.80, 4.36)
Sex of respondent						
Female	98 (20.0)	1.0	1.0	76 (16.2)	1.0	1.0
Male*	46 (28.4)	1.57 (1.05, 2.39)	1.64 (1.08, 2.51)	36 (23.7)	1.60 (1.02, 2.51)	1.32 (0.81, 2.13)
Marital status						
Single/separated/widowed	25 (17.4)	1.0	1.0	76 (33.2)	1.0	1.0
Married/in a relationship*	119 (82.6)	2.82 (1.77, 4.53)	1.70 (1.15, 2.50)	33 (9.4)	0.21 (0.13, 0.33)	0.29 (0.18, 0.45)
Education level						
Primary	25 (15.9)	1.0		27 (17.0)	1.0	
Secondary	45 (18.1)	1.16 (0.68, 1.99)		52 (20.3)	1.25 (0.74, 2.09)	
Higher and above	74 (30.2)*	2.28 (1.37, 3.82)		32 (15.8)	0.91 (0.52, 1.60)	
Age difference with partner						
5 years or less	76 (27.9)	1.0		21 (9.7)	1.0	
6 years or more	31 (23.5)	0.79 (0.49, 1.28)		10 (9.0)	0.92 (0.42, 2.04)	
No partner*	37 (14.9)	0.45 (0.29, 0.71)		81 (27.7)	2.58 (2.10, 6.10)	
Religion of respondent						
Born again (Pentecostal)	25 (19.1)	1.0		28 (20.9)	1.0	
Catholic	46 (20.9)	1.12 (0.65, 1.93)		38 (17.9)	0.83 (0.48, 1.43)	
Protestant	49 (24.1)	1.35 (0.78, 2.32)		33 (17.7)	0.81 (0.64, 1.42)	
Muslim	24 (25.3)	1.43 (0.76, 2.71)		13 (15.5)	0.69 (0.33, 1.43)	
Discussed with health workers about pregnancy, contraception, birth spacing						
No	14 (14.6)	1.0		12 (12.8)	1.0	
Yes	130 (23.4)	1.79 (0.99, 3.26)		100 (19.0)	1.60 (0.84, 3.06)	
Discussed with partner about childbearing*						
No	45 (14.8)	1.0		87 (25.1)	1.0	
Yes	99 (28.5)	2.29 (1.54, 3.41)		25 (9.1)	0.30 (0.18, 0.49)	
H-Lemeshow statistic			77.9%			81.8%
P value			0.221			0.779

Adjusted for age gender and marital status.

## Discussion

In this population, contraceptive use among the HIV-infected young people was significantly lower than that among HIV-negative. Less than a quarter of the young people sustained contraceptive use throughout the year, and a quarter of the HIV infected never initiated contraception. The male condom was the most common type of contraception used in both groups but the HIV-infected were less likely to use condoms compared to the HIV-negative. The proportions became even smaller when we considered consistent condom use. Only 24% of the sexually active HIV-infected used condoms consistently compared to 38% among the negative group. Very few young people, less than one quarter among the HIV-negative and less than one fifth among the HIV-infected, continued to use contraception throughout the year. Furthermore 12% among the negative and 28% among the HIV-infected did not start contraceptive use at all.

While our finding are similar to other studies in indicating that condoms are the preferred contraceptive method [22], there are few studies that compare contraceptive use among HIV-infected and negative young people. Studies among adults show that the HIV-infected had a higher unmet need for contraception and were less likely to use contraception and condoms compared to their negative counterparts [23,24]. A study from Eastern Uganda reported that HIV-infected people who knew their HIV status were three times or more likely to use a condom at their last sexual activity compared with those who did not know their status [25]. Postulated reasons for inconsistent or no condom use by young people may include infrequent sexual activity and changing to new relationships, causal partnerships, use of alcohols or drugs, and not having condoms at the time of sex [14,26,27].

We show that the young singles were less likely to use condoms, or initiate or sustain contraceptive use than married young people. It was not clear whether this ambivalence about pregnancy prevention was because of infrequent sexual activity as suggested by a study among women from the USA [26], inability to plan and control the next sexual encounter or fear of losing the new partners. However, infrequent sexual activity does not stop a pregnancy or HIV transmission from occurring.

Studies report a lack of safe sex negotiation, gender and power relations and the adolescent's ability to communicate to their partner about contraception as important factors that influence contraceptive use [28-30]. One study reported that adolescents who were unfamiliar with the partner were less likely to use contraception [31]. This is contrary to what is known that once a relationship is more intimate, condom use is stopped [27]. Another drawback to contraceptive use could be the type of contraceptive method young people prefer to use. The male-controlled condom needs commitment from the male partner to be effectively and consistently used.

Age was an important factor in consistency of contraceptive and condom use. Young people over 20 years of age were two times more likely to use contraception than those younger. Moreover, the older ones were almost twice as (OR 1.73) likely to take-up contraception and sustain its use throughout the one-year's follow-up. Researchers have reported an increase in births among 15 -19 year-old HIV-infected adolescent women, suggesting that these women could have an unmet need for contraception [32,33]. A possible explanation would be that young people in the older age group are better able to negotiate for contraceptive use with partner. Furthermore, the fear of HIV acquisition was reported to be the reason for increased condom use among young people [22,34]. Nonetheless, other studies report that young people do not to use condoms for either contraception or HIV prevention because they reduce sexual pleasure and male potency [1,35,36]. These findings have enormous implications for pregnancies and HIV transmission since it may take extra ordinary will-power to initiate condom use if the man believes that he will be impotent [1]. Even so, this does not explain the differences in condom use among the infected and negative because it would have affected both. However, our data shows that the HIV-infected group believed the condom was less effective in reducing HIV transmission than the HIV-negative group. A higher trust in condom effectiveness could have also led to a larger proportion of the HIV-negative young people using condoms compared to the HIV-infected group.

During the one-year follow-up, there were no differences as regards stopping contraceptive use in both groups. However, more of the HIV-negative group compared to the HIV-infected group started using contraception. Being older (20 years or more), in a relationship, having discussed with partner about childbearing, and having higher-level education were associated with the taking up of contraception. Our earlier study reported that HIV-infected young people were unable to negotiate for their fertility preferences for fear of abandonment since they had nowhere else to turn to [37].

Our data show that the HIV-infected group had discussed more with the health workers about pregnancy, birth spacing and HIV prevention issues than the HIV-negative had. This may reflect the challenges the HIV-infected have about child bearing. Conversely, it could also indicate that health workers are giving information about reproduction specifically to the HIV-infected. However, unfortunately, these discussions were not associated with an increase in contraceptive uptake among the infected group. Studies report ambivalence among health workers about childbearing among HIV-infected people [38,39]. Thus, these findings could be a reflection of the way the health workers counsel the HIV-infected young people about reproductive choices leading to non-responsive uptake of such messages. This is particularly the case, if the health workers do not use such encounters to discuss and understand the situation of the young people so as to assist them to make responsible fertility choices and adopt the correct contraceptive use.

The study had some limitations. The study population was selected from those that sought care at health units and cannot be generalised to the general population. These young people may have different personal and behavioural characteristics since they have sought health care and received some counselling and education about reproduction and HIV prevention. Moreover, the low contraceptive use among this population would suggest that it might be even lower among the general population. In addition, we excluded the newly tested and those that did not know their HIV status, so as to see the affect of knowing one's own HIV status on fertility decision-making and sexual risk behaviour. This, however, could have introduced some bias and did not allow for a comparison of contraceptive use between those who knew their HIV status and those who did not know their status. The results may not be generalised to the whole population. In addition, the twelve-month follow-up period was possibly too short and thus any differences we see could be temporary. A longer follow-up could give a better picture. Finally, the information collected was based on recalling events such as the time of starting or stopping contraceptive use. Participants were not able to accurately remember which day they started or stopped using contraception. We overcame this by taking blocks of six months. This may have led to inaccuracy since we could have recorded those who had used contraception for a shorter time as having used it for the whole six months.

## Conclusions

Sexually active HIV-infected young people in Kampala are less likely to use condoms and contraception than their HIV-negative counterparts and this has significant implications for pregnancy and HIV transmission. To minimize HIV transmission and unwanted pregnancies among young people, a comprehensive approach to frame their sexuality is urgently needed. The fact that this group are in care settings but are not using contraception consistently has both public and policy implications. The current dialogue with health workers is not leading to an increase in contraceptive use, especially among the HIV-infected; therefore, health workers should be trained to communicate effectively with young people.

Mobilizing more political commitment and resources, strengthening family planning services and looking for new innovations to re-market condom use are critically needed. Policy and guidelines that empower health workers with resources, information, skills and sensitivity to embrace harm reduction techniques to help young people exercise their sexual and reproductive rights while still practicing safe sex should urgently be developed. One possibility is to review the reproductive health counselling content to ensure that young people receive counselling that stresses the importance of consistent condom use as a means of preventing both pregnancy and HIV transmission.

## Competing interests

None declared. The views expressed here are those of the authors and do not necessarily represent their institutions of affiliation.

## Authors' contributions

JBK and FMM conceived the idea, all authors participated in the design of the study, and JBK did data collection. Data management was done by JBK and FK who were assisted by research assistants and a statistician, and produced the initial draft of results. All authors contributed to data analysis and interpretation. All authors read and approved the final version of the manuscript.

## Pre-publication history

The pre-publication history for this paper can be accessed here:

http://www.biomedcentral.com/1471-2334/11/144/prepub
